# A Novel Barium Complex of a Phenoxazinone Sulfonate Dye

**DOI:** 10.1063/4.0000835

**Published:** 2025-10-27

**Authors:** Philip J Squattrito, Jared T. Doney, Christopher G. Gianopoulos

**Affiliations:** 1 Central Michigan University, Mount Pleasant, Michigan, USA; 2 University of Toledo, Toledo, Ohio, USA

## Abstract

A reaction of 3-amino-4-hydroxybenzenesulfonic acid and barium hydroxide in water has yielded an unexpected product, a bis(2-amino-3-oxo-3H-phenoxazinone-8-sulfonato)tetraaquabarium complex Ba(O_3_SC_12_H_7_N_2_O_2_)_2_(H_2_O)_4_. The compound crystallizes as small red needles in the triclinic space group *P*1-bar with two formula units in the cell. The barium cation is 8-coordinate with Ba–O distances to three sulfonate O atoms, one carbonyl O atom, and four water molecules ranging from 2.716(2) to 2.807(1) Å. The structure is built of layers of nearly parallel 2-amino-3- oxo-3H-phenoxazinone-8-sulfonate anions (interplanar separation ca. 3.5 Å) alternating with layers of the hydrated barium cations. The packing is further reinforced by O–H…O hydrogen bonds between coordinated water molecules and sulfonate O atoms. We subsequently learned that the condensation reaction that converted 3-amino-4-hydroxybenzenesulfonic acid to 2-amino-3-oxo-3H-phenoxazinone-8-sulfonic acid was reported to occur via an enzymatic pathway involving a fungus (Forte *et al.*, 2010). The compound belongs to a large class of compounds that are of interest as dyes and are the subject of a US patent application. The barium complex was a trace product of our reaction and it is presently unknown whether the 2-amino-3-oxo-3H-phenoxazinone-8-sulfonic acid is present in the thirty year old 3-amino-4-hydroxybenzenesulfonic acid reagent or formed *in situ* during the reaction.
